# Editorial: Immune regulatory functions of biologically active compounds from fungi

**DOI:** 10.3389/fimmu.2023.1147777

**Published:** 2023-02-07

**Authors:** Di Wang, Zhiheng He, Yu Li, Ning Wang

**Affiliations:** ^1^ School of Life Sciences, Jilin University, Changchun, China; ^2^ Engineering Research Center of Chinese Ministry of Education for Edible and Medicinal Fungi, School of Plant Protection, Jilin Agricultural University, Changchun, China; ^3^ School of Medicine, Wake Forest University, Winston-Salem, NC, United States; ^4^ School of Chinese Medicine, The University of Hong Kong, Hong Kong, Hong Kong SAR, China

**Keywords:** Fungi, bioactive compound, immunoregulatory, molecular mechanism, disease

Recently, increasing attention has been paid to the study of edible mushrooms with medicinal functions. In addition to moisture, mushrooms consist of carbohydrates, proteins, fats, minerals, and nucleic acids, which have various pharmacological effects (1). The immune system plays an indispensable role in human health and disease. An imbalance of the immune system leads to disease onset and progression. Mushrooms such as *Ganoderma (* 2), *Lentinula edodes (* 3), and *Irpex lacteus (* 4) exhibit immunoregulatory properties that have great potential for therapeutic applications. Although active compounds from mushrooms play roles in certain well-known signaling pathways involved in immune regulation, their structure, mechanisms of action, and target cells/molecules remain unclear. This special issue aims to identify structure-explicit immunomodulatory compounds purified from mushrooms and to explore their immune regulatory mechanisms, including their mechanisms of action and targets, such as immune cells, thus promoting the modern development of the medicinal translation of mushrooms.

Fungal polysaccharides have increasingly become a research hotspot in screening for pharmacological agents. Jiang et al. purified a water-soluble glucan from *Grifola frondosa* (GFPA), identified its structure, and explored its anti-obesity effects in high-fat diet (HFD)-fed mice. GFPA markedly blunted steatosis and fat accumulation and inhibited inflammatory infiltration in both white adipose tissue and the livers of HFD-treated mice. Further studies confirmed that the anti-obesity effect of GFPA is related to the suppression of chronic inflammation *via* Toll-like receptor 4/nuclear factor kappa-B signaling. Wang et al. extracted a homogeneous branched β-1,6-glucan from *Pleurotus eryngii* and found that it promoted the proliferation of immune regulatory cells, including splenic lymphocytes, natural killer cells, and peritoneal cavity phagocytes, and regulated the microbiota composition. These studies have broadened the therapeutic applications of fungal polysaccharides.

Proteins are major regulatory mediators of the immune system. Xu et al. comprehensively compared the immunoregulatory activity of proteins obtained from 13 precious mushrooms and nine common mushrooms. They reported that all of these proteins increased the number of M1-like mouse macrophages by promoting the production of pro-inflammatory cytokines, thus strengthening the function of the immune system. Additionally, they confirmed that the immunoregulatory effect was greater for proteins from precious mushrooms than those from common mushrooms. This study provides direct experimental evidence to confirm the high nutritional value of precious mushrooms.

In addition to carbohydrates and proteins, other bioactive compounds from fungi have shown promising pharmacological performance. Wang et al. found that piceatannol, a dietary polyphenolic compound, is a promising enhancer of immunogenic cell death that efficiently improves the therapeutic effect of chemotherapeutics, such as oxaliplatin, by activating autophagy. Indole compounds, such as L-tryptophan and its derivatives, are widely distributed in mushrooms and have been reported to regulate immunological function (5). 3-Indolepropionic acid (IPA) is a tryptophan metabolite that maintains homeostasis in the intestinal environment in a steatohepatitis rat model (6). Fu et al. further explored the pharmacological effects of IPA on dextran-sodium-sulfate-induced colitis in mice. The results demonstrated that IPA significantly improved colitis-like symptoms and regulated the expression of genes involved in immune-related pathways. Further microbiota analysis suggested that the increase in the expression levels of gut immune-related genes was positively linked to the relative abundance of probiotics, such as *Alloprevotella* and *Catenibacterium*. More preclinical models and various methods should be used to confirm the activities of these small molecules before their translation into clinical therapy.

In summary, these studies have confirmed the pharmacological effects of bioactive compounds extracted from mushrooms and explored the underlying mechanisms. These findings provide experimental evidence and theoretical support for the clinical application of fungal bioactive components, accelerating the development of mushroom-based translational medicine.

## Author contributions

All authors listed have made a substantial, direct, and intellectual contribution to the work and approved it for publication.
